# USP7 mediates pathological hepatic de novo lipogenesis through promoting stabilization and transcription of ZNF638

**DOI:** 10.1038/s41419-020-03075-8

**Published:** 2020-10-10

**Authors:** Wenkai Ni, Shengli Lin, Saiyan Bian, Wenjie Zheng, Lishuai Qu, Yihui Fan, Cuihua Lu, Mingbing Xiao, Pinghong Zhou

**Affiliations:** 1 Endoscopy Center and Endoscopy Research Institute, Zhongshan Hospital, Fudan University, No. 180, Fenglin Road, 200032 Shanghai, People’s Republic of China; 2 Department of Gastroenterology, Affiliated Hospital of Nantong University, No. 20, XiSi Road, 226001 Nantong, People’s Republic of China; 3 Medical College, Nantong University, No.19, Qixiu Road, 226001 Nantong, People’s Republic of China; 4 Research Center of Clinical Medicine, Affiliated Hospital of Nantong University, No. 20, XiSi Road, 226001 Nantong, People’s Republic of China

**Keywords:** Cancer metabolism, Mechanisms of disease

## Abstract

Aberrant de novo lipogenesis (DNL) results in excessive hepatic lipid accumulation and liver steatosis, the causative factors of many liver diseases, such as non-alcoholic fatty liver disease (NAFLD), non-alcoholic steatohepatitis (NASH), and hepatocellular carcinoma (HCC). However, the underlying mechanism of DNL dysregulation remains largely unknown. Ubiquitination of proteins in hepatocytes has been shown to be widely involved in lipid metabolism of liver. Here, we revealed that Ubiquitin-specific peptidase 7 (USP7), a deubiquitinase (DUB), played key roles in DNL through regulation of zinc finger protein 638 (ZNF638) in hepatocytes. USP7 has been shown not only to interact with and deubiquitylate ZNF638, but also to facilitate the transcription of ZNF638 via the stabilization of cAMP responsive element binding protein (CREB). USP7/ZNF638 axis selectively increased the cleavage of sterol regulatory element binding protein (SREBP1C) through AKT/mTORC1/S6K signaling, and formed USP7/ZNF638/SREBP1C nuclear complex to regulate lipogenesis-associated enzymes, including acetyl-CoA carboxylase (ACACA), fatty acid synthase (FASN), and Stearoyl-CoA desaturase (SCD). In the mice liver steatosis model induced by fructose, USP7 or ZNF638 abrogation significantly ameliorated disease progression. Furthermore, USP7/ZNF638 axis participated in the progression of lipogenesis-associated HCC. Our results have uncovered a novel mechanism of hepatic DNL, which might be beneficial to the development of new therapeutic targets for hepatic lipogenesis-associated diseases.

## Introduction

Lipid metabolism is controlled by a complicated network of signaling and enzymatic events in multiple cell types, by which it fulfils the requirements of energy storage and consumption of the body, membrane regeneration of the cell, as well as cellular homeostasis^1^. De novo lipogenesis (DNL) that converts acetyl-CoA subunits to fatty acids, following by incorporation into triglycerides (TGs) for energy storage, is a highly regulated metabolic pathway. Deregulation of DNL in the major lipogenic tissues has been observed to be relevant to various metabolic diseases, such as obesity, diabetes, and metabolic syndrome. In the liver, aberrant DNL characterized by excessive hepatic triglyceride deposition is considered to be the hallmark of non-alcoholic fatty liver disease (NAFLD) and non-alcoholic steatohepatitis (NASH)^2^. Overdose intake of carbohydrate (fructose, glucose) is one of important causes of aberrant hepatic DNL that leads to triglyceride accumulation in the livers of NAFLD patients^3^. The hepatic steatosis, accompanied by chronic inflammation, is regarded as a key risk factor of cirrhosis that will progress to end-stage liver diseases, including hepatocellular carcinoma (HCC) if without proper clinical interference^4^. Therefore, unveiling the mechanisms of abnormal DNL is critical for understanding the pathogenesis of DNL-associated diseases.

DNL initiates from the conversion of citrate to acetyl-CoA by ATP-citrate lyase (ACLY), which is further turned into malonyl-CoA with the assistance of acetyl-CoA carboxylase (ACACA). Then, the fatty acid synthase (FASN) condenses malonyl-CoA to produce palmitate, which is then generated to monounsaturated fatty acids serving as the substrate of Stearoyl-CoA desaturase (SCD)^5^. So, the dynamic regulation of these synthases has been highlighted in the processes of DNL that may contribute to the pathogenesis of NAFLD, NASH, and HCC in conditions of abnormity. For example, mutations of AMP-activated protein kinase (AMPK) phosphorylation sites at ACACA1 (S79A) and ACACA2 (S212A) display an abnormal hepatic lipogenesis in mice that are prone to tumor formation^6^. Also, ablation of FASN has been found to significantly ameliorate AKT-driven hepatocarcinogenesis^7^. As such, several small molecular inhibitors targeting these enzymes have been successfully developed and manifest noteworthy therapeutic effects on NAFLD, NASH, and HCC^8,9^. However, the complicated network of DNL might hint diverse targets for the design of better strategies to treat metabolic diseases.

Regulation of protein ubiquitination has been proved to mediate many cellular events, including cell cycle, apoptosis, and other physiological functions^10^. Once the ubiquitin is enzymatically activated and binds to the targeting protein, degradation of the substrate is proceeded by 26S proteasome^11^. Deubiquitinases (DUB) reversely catalyze such degradable process through which to ensure protein stabilization^12,13^. Ubiquitin-specific peptidase 7 (USP7), the seventh identified DUBs^14^, is a robust tumor enhancer due to its deubiquitination of N-Myc^15^ or MDM2 that results in the inactivation of P53^16^. Consequently, the USP7-specific antagonist P22077 shows prominent anti-cancer effects in a manner of P53 dependent or independent pathway^17,18^. In the liver, the dysregulation of USP7 has been observed and used for a prognosis of HCC patients^19–21^. A recent finding has linked the regulatory role of USP7 to the hepatic lipid metabolism, showing that USP7 acts as a PiT1-binding partner to regulate hepatic lipogenesis through influence of glucose metabolism^22^. These observations indicate that USP7 probably participates hepatic pathogenesis through mediation of lipogenesis. However, the regulatory mechanism of USP7 by deubiquitination in DNL remains unclear.

Zinc finger protein 638 (ZNF638) belongs to zinc finger proteins, which constitute the largest transcription regulator superfamily. In early adipogenesis, ZNF638 binds to CCAAT/enhancer-binding protein (C/EBP) β and C/EBPδ, leading to the transcriptional activation of PPARγ, which in turn controls differentiation of adipocytes^23^. However, little information is available about the role of ZNF638 in lipogenesis. In the present study, we have demonstrated that ZNF638 acted as a novel substrate of USP7 to mediate DNL. USP7 controls the protein level of ZNF638 in the hepatocytes through deubiquitination and transcriptional activation via cAMP responsive element binding protein (CREB). Also, the two proteins promote nuclear accumulation of cleaved-SREBP1C via AKT/mTORC1/S6K-dependent and independent manners, by which USP7 facilitates DNL through activation of ACACA, FASN, and SCD. Our study has provided a novel mechanism of USP7 regulation on the hepatic lipogenesis, which might be beneficial for the therapeutic development of the lipogenesis-associated liver diseases.

## Results

### USP7 interacts with and deubiquitinates ZNF638 in various hepatoma cells

**Fig. 1:**
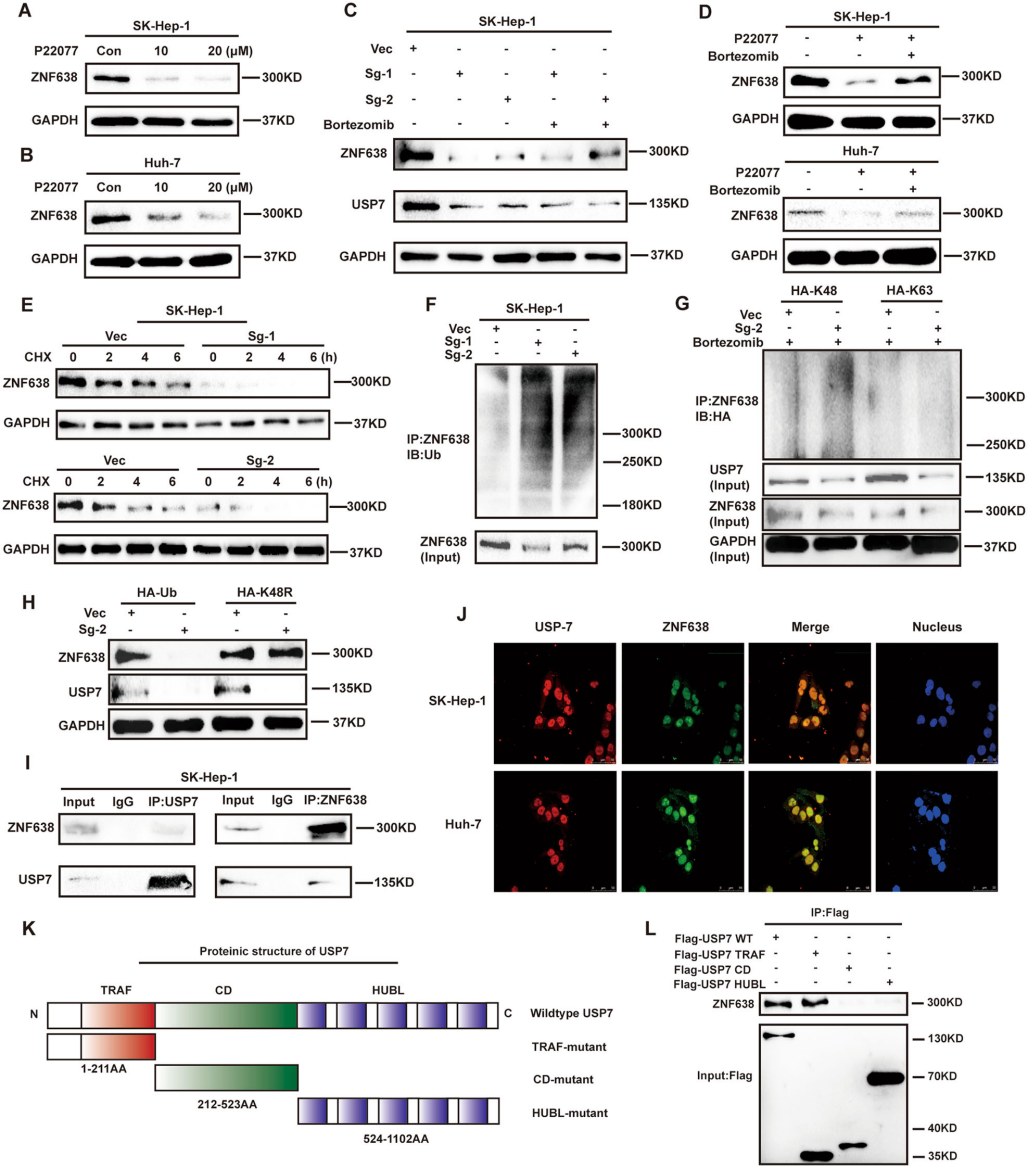
USP7 stabilizes ZNF638 protein partially through its deubiquitinating activity. **A**, **B** ZNF638 protein level was down-regulated with the treatment of P22077 in SK-Hep1 and Huh-7 cells. **C** The knockdown efficiency of USP7 by two specific SgRNA (Sg1, Sg2) and their effects on ZNF638 expression were determined by immunoblotting in SK-Hep1 cells; proteasome inhibitor Bortezomib (100 nM 8 h) partially rescued declined expression of ZNF638 in USP7-deficient cells. **D** P22077 (20 μM 24 h) induced ZNF638 protein inhibition was partially rescued by Bortezomib (100 nM 8 h) in SK-Hep1 and Huh-7 cells. **E** The half-life of ZNF638 protein in normal and USP7-deficient SK-Hep1 cells was determined by using CHX (100 μg/ml) at indicated time points. **F** Genetic inhibition of USP7 in SK-Hep1 cells increased ubiquitination level of ZNF638. **G** The enhanced polyubiquitination of ZNF638 in SK-Hep1 cells according to USP7 knockdown was mainly Lys48 but not Lys 63 linked polyubiquitination. **H** K48-resistant ubiquitin (Lys48 to Arg48) could reverse ZNF638 expression in USP7-deficient SK-Hep1 cells. **I** ZNF638 and USP7 were endogenously interacted with each other in SK-Hep1 cells as determined by Co-IP assay. **J** USP7 and ZNF638 were mainly co-localized in cell nucleus as determined by immunofluorescence in Huh-7 and SK-Hep1 cells. **K** Schematic illustration of the protein structure of USP7. **L** Three of flag tagged truncated mutant plasmids (Flag-TRAF, Flag-CD, and Flag-HUBL) and wild-type USP7 (Flag-USP7) were transfected into SK-Hep1 cells, followed by immunoprecipitation using flag antibody and immunoblotting with ZNF638 antibody. Each immunoblotting assay was performed at least three times from independent studies.

To understand the regulatory relationship between USP7 and ZNF638, we inhibited the activity of USP7 in the SK-Hep1 or Huh-7 hepatoma cell line by addition of pharmacological inhibitor P22077 and then observe the protein level of ZNF638. Results showed that inhibition of USP7 by P22077 significantly reduced the protein level of ZNF638 (Fig. 1A, B). Next, we knocked down the expression of USP7 in SK-Hep1 cells by two specific SgRNAs (Fig. 1C). As a consequence, knockdown of USP7 remarkably repressed the expression of ZNF638. Such effect on ZNF638 of USP7 interference was partially rescued by proteasome inhibitor, Bortezomib (Fig. 1C, D). Conversely, overexpression of USP7 was able to up-regulate the expression of ZNF638 in SK-Hep1 cells (Supplementary Fig. S1A). These results indicate that ZNF638 is a potential substrate that can be stabilized by USP7. To further verify these results, we examined the half-life of ZNF638 in USP7-deficient cell lines. As expected, the half-life of ZNF638 in absence of USP7 was apparently shorter compared with vector cells (Fig. 1E and Supplementary Fig. S1B). USP7 primarily regulates substrate proteins by deubiquitination. Thus, we examined the ubiquitination of ZNF638 following inhibition or genetic deletion of USP7. Results showed that depletion or inhibition of USP7 significantly increased the ubiquitination of ZNF638 (Fig. 1F and Supplementary Fig. S1C). Co-transfection of mutant HA-Ub containing only one lysine at position 63 (Lys63) or 48 (Lys48) in SK-Hep1 cells revealed that polyubiquitin chain on ZNF638 regulated by USP7 was mainly Lys48-linked (Fig. 1G). Transfection of K48-resistant ubiquitin (Lys48 to Arg48 mutant, K48R) reversed the expression of ZNF638 in USP7-depleted SK-Hep1 cells, suggesting the Lys48-linked polyubiquitin chain on ZNF638 was under regulation of USP7 (Fig. 1H). Immunoprecipitation was performed to address the interaction between USP7 and ZNF638 in SK-Hep1 cells. As shown in Fig. 1I, ZNF638 was present in the USP7-associated complex immunoprecipitated with anti-USP7 antibody, so was USP7 in the ZNF638-associated complex. Similar results were displayed by using Huh-7 cells (Supplementary Fig. S1D). Immunofluorescence showed that USP7 and ZNF638 co-localized in the nucleus (Fig. 1J). To explore the specific domain of USP7 that binds to ZNF638, we established three truncated mutants of USP7, with different domains including the N-terminal TRAF domain (TRAF), the central catalytic domain (CD) as well as the C-terminal regulatory domain composed of five ubiquitin-like repeats (HUBL) (Fig. 1K)^24^. FLAG-USP7 full length or deletion mutant proteins were immunoprecipitated from cell lysates and immunoblotted with ZNF638 antibodies. In this assay, we found that USP7 full length and TRAF domain but not CD and HUBL domain were able to pull down ZNF638, indicating TRAF domain mediates the association between USP7 and ZNF638 (Fig. 1L). Taken together, USP7 is able to positively regulate protein level of ZNF638 through deubiquitination.

### USP7 transcriptionally regulates the expression of ZNF638 via stabilization of CREB

**Fig. 2:**
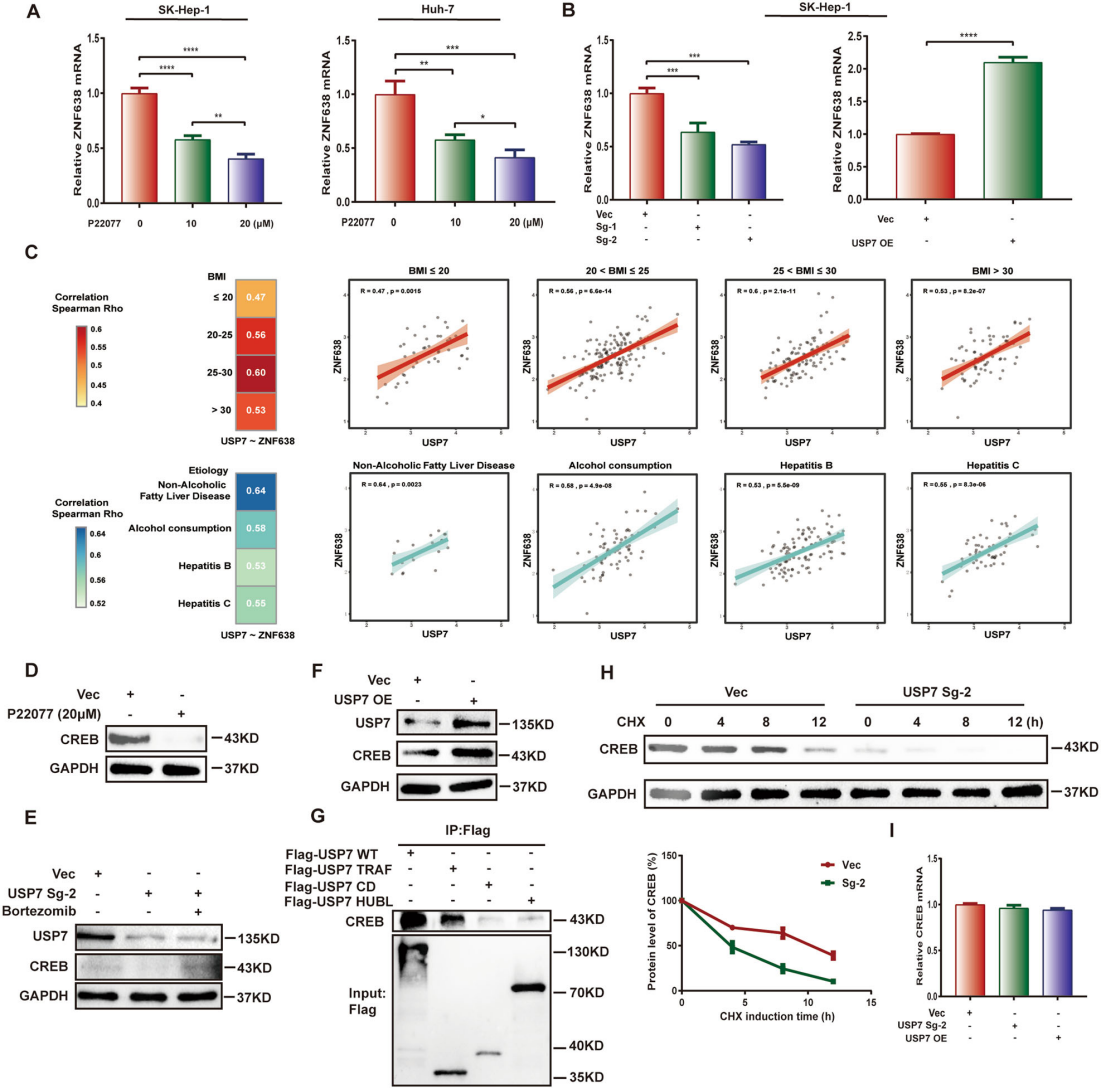
USP7 positively regulates the mRNA of ZNF638. **A** P22077 treatment caused concentration-dependent reduction of ZNF638 mRNA in SK-Hep1 cells and Huh-7 cells. **B** USP7 knockdown or overexpression resulted in paralleled decrease or increase of ZNF638 mRNA in SK-Hep1 cells. **C** The mRNA of USP7 and ZNF638 were positively correlated in HCC patients based on the data from TCGA; the correlation Rho values differed among the different BMI and etiology groups. **D** CREB protein was suppressed via 20 μM of P22077 induction in SK-Hep1 cells. **E** CREB protein was reduced in USP7-deficient SK-Hep1 cells, while proteasome inhibitor Bortezomib (100 nM 8 h) rescued the effects. **F** Overexpression of USP7 in SK-Hep1 cells resulted in the enhanced protein level of CREB. **G** Flag tagged USP7 wild-type, TRAF domain, CD domain, HUBL domain were transfected in SK-Hep1 cells, followed by immunoprecipitation to determine the binding site of USP7 to CREB. **H** The half-life of CREB protein in normal and USP7-deficient SK-Hep1 cells was determined by using CHX (100 μg/ml) at indicated time intervals. **I** The mRNA levels of CREB in SK-Hep1 cells were unchanged neither by USP7 knockdown nor overexpression. The representative statistical results were performed using one-way ANOVA or unpaired *t-*test and shown as means ± SEM from three independent experiments. *p < 0.05, **p < 0.01, ***p < 0.001, ****p < 0.0001.

Besides USP7 regulates the protein level of ZNF638, we also examined whether USP7 regulates the mRNA level of ZNF638. Interestingly, incubation of P22077 remarkably decreased the mRNA levels of ZNF638 in both SK-Hep1 and Huh-7 cells in a dose-dependence (Fig. 2A). The similar effect was also shown in USP7-deficient SK-Hep1 cells, and overexpression of USP7 conversely elevated the mRNA of ZNF638 (Fig. 2B). We further analyzed RNA sequencing data in TCGA database to take an insight into the correlation between USP7 and ZNF638 at mRNA levels in HCC patients. Results demonstrated positive correlation between expression of USP7 and ZNF638 (Fig. 2C). The Rho value rose up in patients with higher BMI index, or with specific pathological types related to lipid metabolic disorders, such as alcoholic liver disease or NAFLD (Fig. 2C). These results suggest that USP7 not only stabilizes ZNF638 protein but also up-regulates ZNF638 mRNA. To elucidate the potential mechanism by which USP7 positively regulates the mRNA of ZNF638, we assessed CREB, a known transcription factor of ZNF638^25^ with induction or inhibition of USP7 in SK-Hep1 cells. Importantly, the protein of CREB was markedly reduced via USP7 knockdown or P22077 administration, whereas increased by USP7 overexpression (Fig. 2D–F). Bortezomib rescued the protein level of CREB upon USP7 knockdown (Fig. 2E). Co-IP assay confirmed the interaction between USP7 and CREB in SK-Hep1 cells (Supplementary Fig. S2A). The interaction between USP7 and CREB was mainly through the TRAF domain of USP7 (Fig. 2G). Enhanced ubiquitination and declined half-life of CREB protein were also detected in the condition of USP7 knockdown (Supplementary Fig. S2B and Fig. 2H). However, the mRNA level of CREB were not altered no matter USP7 overexpression or knockdown (Fig. 2I). Thus, USP7 is able to promote the transcription of ZNF638 by stabilizing the transcription factor CREB.

### USP7 is involved in DNL via regulation of ZNF638 in vitro

**Fig. 3:**
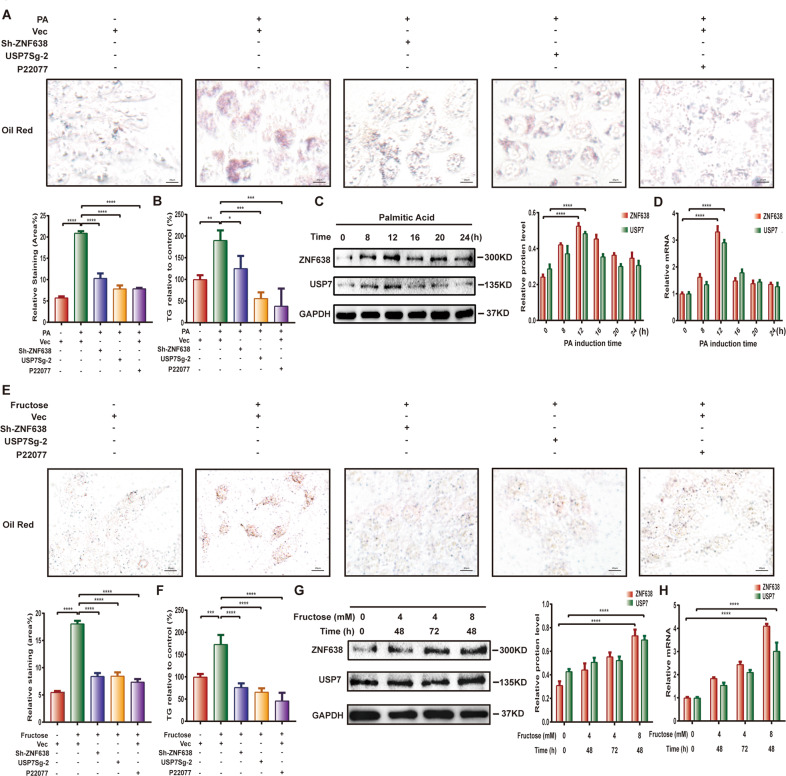
USP7 and ZNF638 are associated with hepatic de novo lipogenesis in vitro. **A** Grouped SK-Hep1 cells as normal, USP7 knockdown, ZNF638 knockdown, and P22077 induction (10 μM pretreated for 24 h) were treated with or without palmitic acid (0.5 mM) for 8 h and applied to oil-red o assay; quantitative analysis of the staining area was revealed by ImageJ software. **B** The relative TG content of SK-Hep1 cells in different groups were measured by TG kit. **C** The alterations of USP7 and ZNF638 protein levels after PA (0.5 mM) induction at different time intervals in SK-Hep1 cells were examined by immunoblotting; quantification of protein levels relative to GAPDH was performed using ImageJ. **D** The alterations of USP7 and ZNF638 mRNA levels upon PA induction at different time intervals were examined by RT-PCR. **E** Grouped SK-Hep1 cells were treated with or without fructose (8 mM) for 48 h and applied to oil-red o assay; quantitative analysis of the staining area was revealed by ImageJ software. **F** The relative TG content of cells in different groups was measured by TG kit. **G** The alterations of USP7 and ZNF638 protein levels within different fructose inducing were assessed by immunoblotting and quantified by ImageJ. **H** The alterations of USP7 and ZNF638 mRNA levels upon different fructose induction were detected by RT-PCR. Representative statistical results were performed using one or two-way ANOVA test and shown as means ± SEM from three independent experiments. *p < 0.05, **p < 0.01, ***p < 0.001, ****p < 0.0001.

To address the physiological function of USP7 in DNL via regulation of ZNF638, we established an in vitro model of lipogenesis using palmitic acid (PA)-induced adipogenic differentiation in SK-Hep1 cells. Following incubation of 0.5 mM PA for 8 h, most of cells began to manifest a high-fat phenotype as detected by Oil-Red O and TG assays (Fig. 3A, B). Whereas knockdown of ZNF638 significantly reduced the lipids accumulation (Fig. 3A). Depletion of USP7 either by SgRNA or by inhibitor P22077 also suppressed the PA-driven effects (Fig. 3A). Accordingly, the triglyceride content was decreased in the cells interfered with USP7 or ZNF638 (Fig. 3B). In addition, PA stimulation induced expression of USP7 and ZNF638 with a peak at 12 h, both at mRNA and protein levels (Fig. 3C, D). To further clarify the role of USP7 and ZNF638 in DNL, the model of fructose-induced lipogenesis was performed. Consistently, treatment of fructose (8 mM) for 48 h remarkably induced lipid accumulation and triglyceride content in SK-Hep1 cells (Fig. 3E, F). Again, such effect could be attenuated by interference of ZNF638 or USP7 (Fig. 3E, F). Addition of fructose also increased the mRNA and protein levels of both USP7 and ZNF638 (Fig. 3G, H). These results indicate that USP7 is involved in DNL via regulation of ZNF638.

### USP7 and ZNF638 mediate hepatic DNL through regulation of ACACA, FASN, and SCD enzymes

**Fig. 4:**
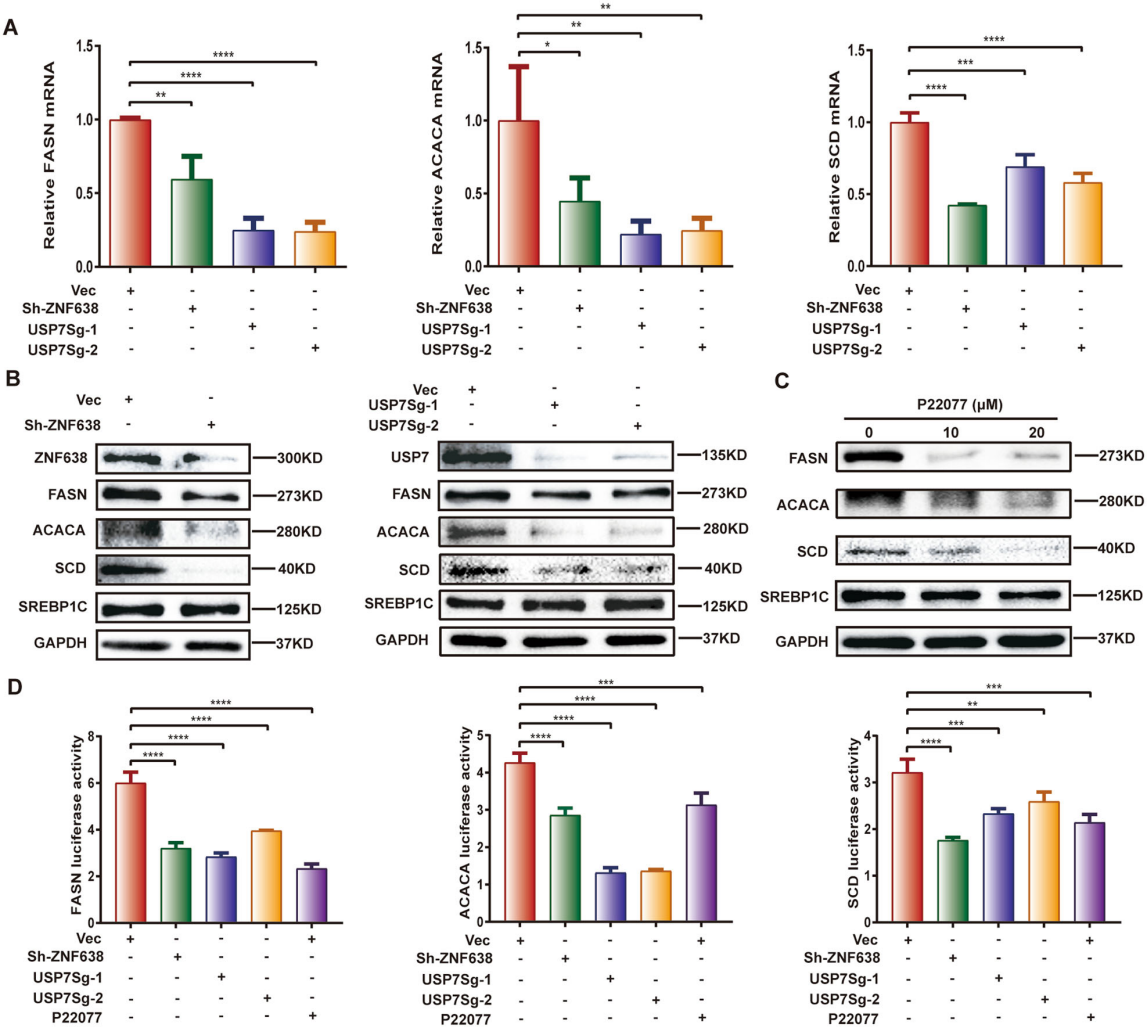
USP7 and ZNF638 transcriptionally regulate DNL effectors. **A** The relative mRNA levels of ACACA, FASN, SCD in normal, USP7-deficient, ZNF638-deficient SK-Hep1 cells were determined by real-time PCR. **B** The protein levels of ACACA, FASN, SCD and SREBP1C in normal, USP7-deficient, ZNF638-deficient SK-Hep1 cells were assessed by immunoblotting. **C** Pharmacological inhibition of USP7 reduced protein levels of ACACA, FASN, and SCD in SK-Hep1 cells. **D** The promoter activity of ACACA, FASN, and SCD relating to ZNF638, USP7 knockdown or P22077 treatment was determined by luciferase reporter assay. The representative statistical results were performed using one-way ANOVA test and shown as means ± SEM from three independent experiments. *p < 0.05, **p < 0.01, ***p < 0.001, ****p < 0.0001.

The enzymes FASN, ACACA, and SCD are key synthases of DNL, whereas their transcription is under control of SREBP1C. To understand whether the regulatory roles of USP7 and ZNF638 in DNL attribute to the activation of lipogenesis-related enzymes, the expression of FASN, ACACA, SCD, and SREBP1C was thus examined. Results showed that the mRNA levels of FASN, ACACA, and SCD were significantly down-regulated following silence of ZNF638 or USP7 in SK-Hep1 cells (Fig. 4A). So did the same changes in the protein levels (Fig. 4B, C). However, the expression of SREBP1C at level of mRNA or protein was unaltered in the same condition (Supplementary Fig. S3A and Fig. 4B, C). Next, we investigated the regulatory roles of USP7 or ZNF638 on the transcription of FASN, ACACA, and SCD by using luciferase reporting plasmids containing promoters of FASN, ACACA, or SCD. Luciferase assays demonstrated that the promoter activity was markedly suppressed in the SK-Hep1 cells after silence of ZNF638 or USP7 (Fig. 4D). In the fructose-induced lipogenesis model, fructose enhanced the expression of FASN, ACACA, SCD, and SREBP1C, however the expression level of FASN, ACACA, SCD but not SREBP1C could be reversed by USP7 or ZNF638 interference (Supplementary Fig. S3B). These results indicate that USP7/ZNF638 axis regulates hepatic DNL through transcriptional activation of FASN, ACACA, and SCD without affecting the full-length protein of SREBP1C.

### USP7/ZNF638 axis promotes cleaved-SREBP1C accumulation in nucleus via AKT-mTORC1-S6K-dependent and -independent manners

**Fig. 5:**
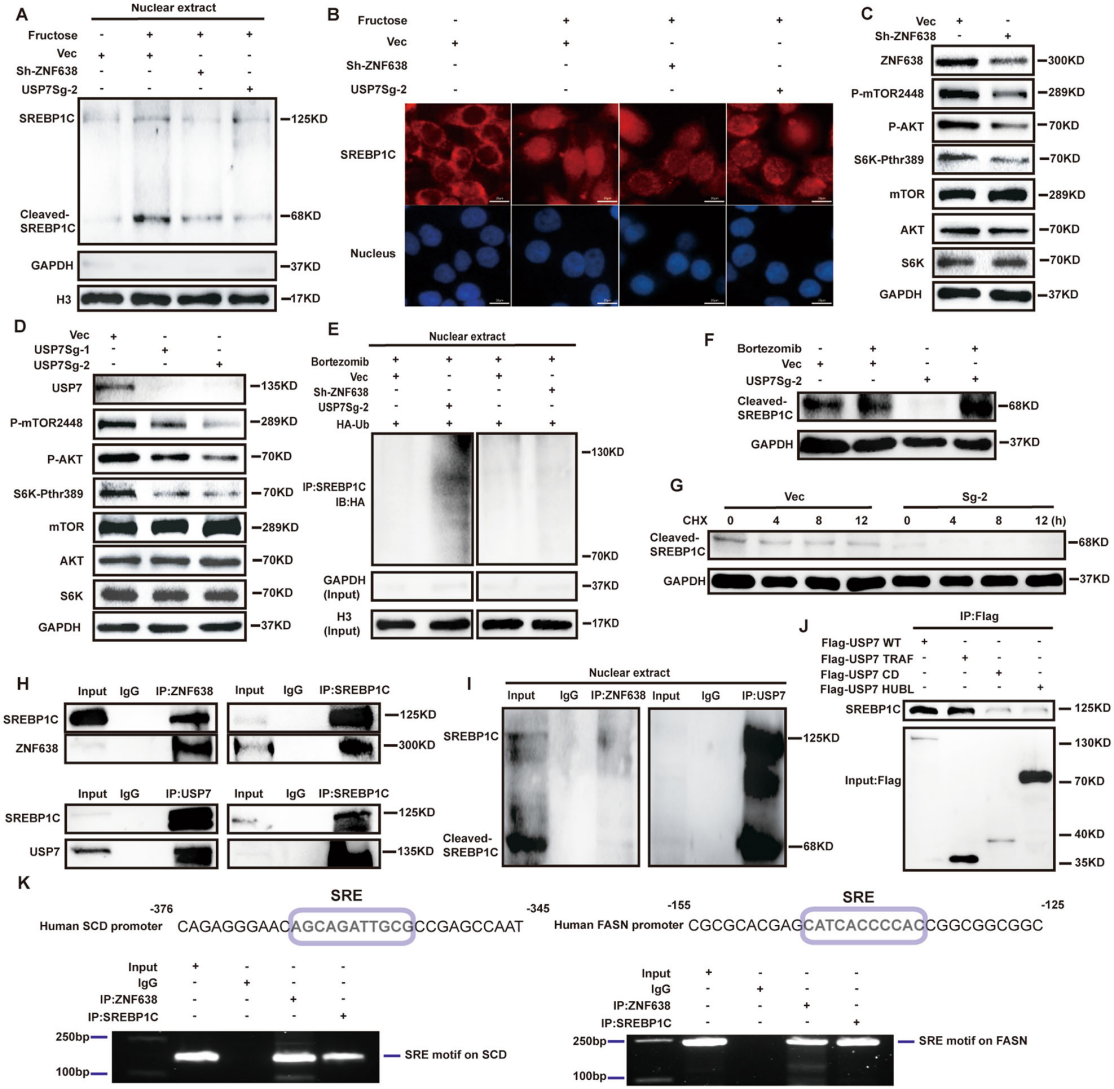
USP7 and ZNF638 control the nuclear accumulation of cleaved-SREBP1C. **A** The effects of USP7 or ZNF638 knockdown on fructose (20 mM 48 h) induced cleaved-SREBP1C were verified by immunoblotting, using nuclear extracts from SK-Hep1 cells. **B** The subcellular location changes of SREBP1C upon the fructose induction (20 mM 48 h) with or without USP7, ZNF638 knockdown were explored by immunofluorescence in SK-Hep1 cells. **C** Genetic ablation of ZNF638 in SK-Hep1 cells reduced phosphorylation levels of mTOR, AKT, and S6K. **D** Genetic ablation of USP7 in SK-Hep1 cells reduced phosphorylation levels of mTOR, AKT, and S6K. **E** USP7 but not ZNF638 knockdown could increase the ubiquitination of cleaved-SREBP1C in SK-Hep1 cells. **F** The decreased cleaved-SREBP1C resulting from USP7 knockdown could be rescued by bortezomib (100 nM 8 h) in SK-Hep1 cells. **G** The half-life of cleaved-SREBP1C in USP7-deficient SK-Hep1 cells was shortened. **H** The reciprocal interactions between ZNF638/USP7 and full-length SREBP1C were observed by Co-IP assays in SK-Hep1 cells. **I** The endogenous interactions between ZNF638/USP7 and cleaved-SREBP1C were determined by Co-IP assays using nuclear lysates of SK-Hep1 cells. **J** The binding sites of USP7 on SREBP1C were determined by transfecting truncated USP7 mutants (Flag-TRAF, Flag-CD, and Flag-HUBL) into SK-Hep1 cells, following immunoprecipitation with flag antibody and detection of SREBP1C. **K** The binding of ZNF638 on the promoters of SREBP1C target genes were examined via ChIP assay in SK-Hep1 cells, lanes of IgG and SREBP1C were set as negative and positive control.

The nuclear translocation of cleaved-SREBP1C is essential for its physiological roles in high-nutrient condition. To ascertain whether USP7/ZNF638 axis regulates the cleavage of SREBP1C in SK-Hep1 cells, we determined the protein level of cleaved-SREBP1C with or without deletion of USP7 and ZNF638. As shown in Supplementary Fig. S4A, knockdown of USP7 or ZNF638 resulted in a significant down-regulation of cleaved-SREBP1C. Also, fructose induced elevation of cleaved-SREBP1C and its nuclear accumulation could be attenuated by knockdown of USP7 or ZNF638 in SK-Hep1 cells (Fig. 5A, B). The data demonstrates that USP7/ZNF638 axis activates SREBP1C through inducing its cleavage.

AKT-mTORC1-S6K pathway has been found to be implicated in the processing and cleavage of SREBP1C. To shed light on the potential regulation of USP7/ZNF638 axis on the pathway, we examined the phosphorylation of mTOR (Ser2448), AKT (Ser473) and S6K (Thr389) following interference of ZNF638 or USP7. Results revealed that the phosphorylation levels of mTOR (Ser2448), AKT (Ser473) or S6K (Thr389) were significantly decreased after depletion of ZNF638 or USP7 in SK-Hep1 cells (Fig. 5C, D and Supplementary Fig. S4B). In the model of fructose-induced DNL in SK-Hep1 cells, phosphorylation of these proteins was remarkably increased, in line with the generation of cleaved-SREBP1C. Treatment of cells with mTOR inhibitor INK128 obviously attenuated such fructose-induced effects (Supplementary Fig. S4C). These results indicate that USP7/ZNF638 axis facilitates SREBP1C cleavage via AKT-mTORC1-S6K pathway.

The cleaved-SREBP1C in nucleus is vulnerable to ubiquitin-dependent protein degradation. We therefore assumed whether USP7 could stabilize the nuclear cleaved-SREBP1C. By immunoprecipitation of SREBP1C from nuclear extracts, we found that the ubiquitination of cleaved-SREBP1C was remarkably increased upon knockdown of USP7 but not of ZNF638 (Fig. 5E). Additional knockdown of ZNF638 in USP7-deficient cells was able to further down-regulate cleaved-SREBP1C without affecting its ubiquitination (Supplementary Fig. S4D). Bortezomib efficiently reversed the proteinic inhibition of cleaved-SREBP1C caused by USP7 interference (Fig. 5F). Accordingly, the half-life of cleaved-SREBP1C also became shorter owing to the loss of USP7 (Fig. 5G and Supplementary Fig. S4E). Co-immunoprecipitation assay from whole cells or nuclear lysates displayed that both ZNF638 and USP7 could bind to the full-length and cleavage form of SREBP1C (Fig. 5H, I). Further domain analysis using truncated USP7 mutants revealed that the TRAF domain of USP7 was essential for the binding of USP7 to SREBP1C (Fig. 5J). Considering USP7/ZNF638 formed a nuclear complex with cleaved-SREBP1C and the identified role of ZNF638 as a transcription cofactor, we further employed ChIP assay to clarify whether ZNF638 could bind to the promoters of the target genes of SREBP1C. By identifying the sterol-responsive element (SRE) on the promoter regions of FASN and SCD, which was recognized by SREBP1C, we proved the binding of both ZNF638 and SREBP1C to the promoters of FASN and SCD (Fig. 5K). These findings indicate that USP7 regulate the abundance of cleaved-SREBP1C through both AKT-mTORC1-S6K signaling and the stability of cleaved-SREBP1C.

### USP7/ZNF638 axis participates in fructose-induced hepatic steatosis in vivo

**Fig. 6:**
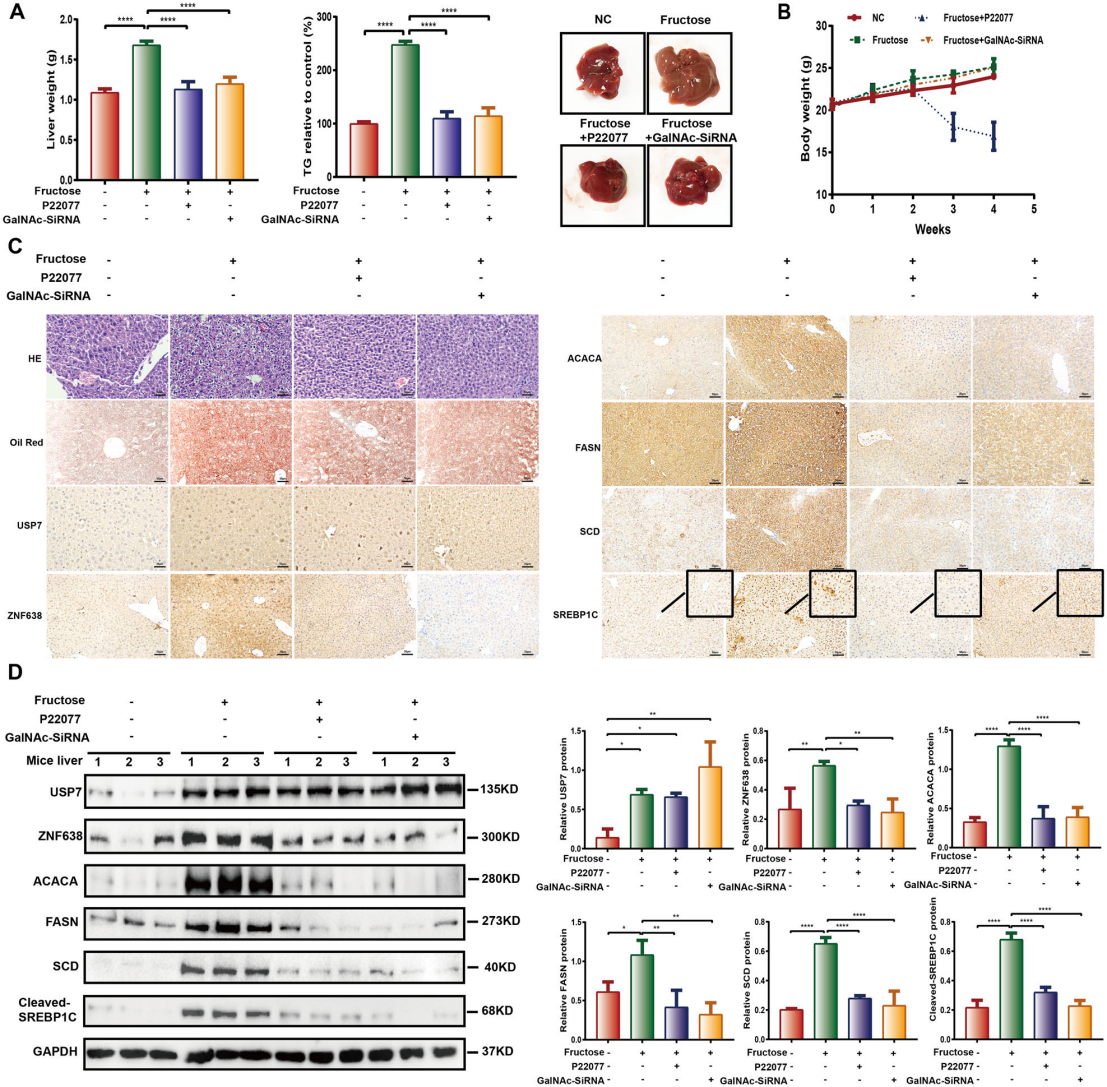
USP7 and ZNF638 participate in fructose-induced hepatic steatosis in vivo. **A**, **B** C57BL/6 mice were fed with standard chow diet or diet added with 30% fructose in drinking water. The fructose group mice were subclassified and disposed to intraperitoneal injection of P22077 (10 mg/kg BW) and GalNAc-ZNF638-SiRNA (2 μg/g BW) every week. The body weight of the mice was weighed every week, and after 4 weeks induction, the liver weight and the TG content of the liver were assessed. **C** The pathological alterations among different groups were detected by HE staining using paraffin sections; the liver lipids accumulation was determined by oil-red o assay; the expression of USP7, ZNF638, ACACA, FASN, SCD, and SREBP1C among different groups were observed by immunochemistry. **D** Alterations of USP7, ZNF638, ACACA, FASN, SCD, and cleaved-SREBP1C among different groups were determined by immunoblotting, followed by quantitative analysis. The representative statistical results were performed using one-way ANOVA test and shown as means ± SEM from three independent experiments. *p < 0.05, **p < 0.01, ***p < 0.001, ****p < 0.0001.

To examine the physiological roles of USP7/ZNF638 axis on DNL in vivo, we established the fructose-induced mouse hepatic steatosis model. The 30% (w/w) of fructose in drinking water slightly increased the body weight (Fig. 6B), but heavy liver steatosis occurred as indicated by the increase in liver weight, TG content, tissue loosen degeneration, and lipids accumulation (Fig. 6A, C). Consistently, levels of USP7 and ZNF638, accompanying with ACACA, FASN, SCD, and nuclear SREBP1C were elevated (Fig. 6C, D). In this model, the N-acetylgalactosamine (GalNAc)-conjugated ZNF638-siRNA (siRNA-1, 2 μg/g BW), which specifically targeted the asialoglycoprotein receptor of hepatocytes, or P22077 (10 mg/kg BW), was intraperitoneal injected every week. The transfection efficiency of CY3 labeled GalNAc-conjugatd siRNA in vivo was confirmed by imaging (Supplementary Fig. S5A) and the knockdown efficiency of ZNF638 in vitro and in vivo was detected by immunoblotting (Supplementary Fig. S5B). The effectiveness of P22077 in vivo was confirmed by decreased hepatic MDM2 level, as it was an important substrate of USP7 (Supplementary Fig. S5C). By four weeks of injection, declined liver weight, TG content and lipids accumulation were observed in both of the experimental groups (Fig. 6A, C). Immunostaining and immunoblotting showed that the fructose-induced ZNF638 and nuclear SREBP1C, as well as DNL enzymes, including ACACA, FASN, and SCD were greatly attenuated upon treatment with GalNAc-conjugated ZNF638-siRNA and P22077 (Fig. 6C, D). The data indicates that USP7/ZNF638 axis mediates aberrant DNL, which is relevant to hepatic steatosis in vivo.

### USP7/ZNF638 axis contributes to lipogenesis-associated hepatic carcinogenesis

**Fig. 7:**
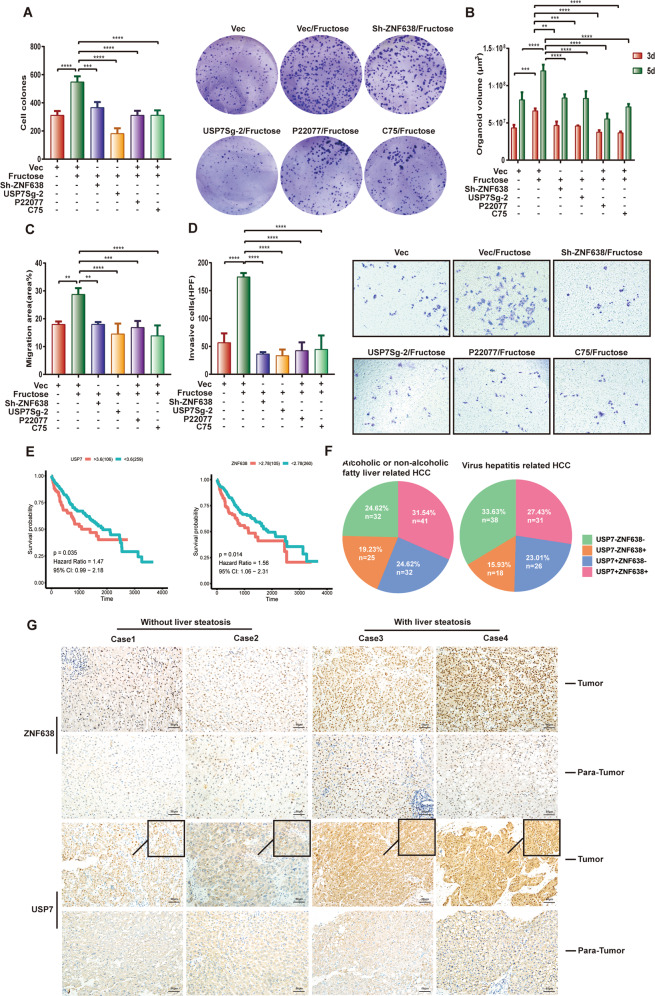
USP7 and ZNF638 affect lipogenesis-associated hepatic carcinogenesis. **A** SK-Hep1 cells with or without stable ZNF638 knockdown, stable USP7 knockdown, P22077 treatment (5 μM), and C75 treatment (1 μg/ml) were cultured with or without fructose (8 mM) for 14 days; after that, the cell clones were harvested and calculated by ImageJ. **B** Seventy percent of different SK-Hep1 cells (ZNF638 knockdown, USP7 knockdown, treatment of 5 μM P22077, treatment of 1 μg/ml C75) and 30% of LXR cells were mixed and glued with Collagen I (5 μg/ml); the culture medium with or without fructose (8 mM) was added with 20 ng/ml b-FGF, 20 ng/ml HGF to promote the growth of both cell lines. At the time points of 3 days and 5 days, organoids volumes of different groups were analyzed. **C** SK-Hep1 cells with or without stable ZNF638 knockdown, stable USP7 knockdown, P22077 treatment (10 μM), and C75 treatment (2.5 μg/ml) were pre-scratched and cultured with or without fructose (8 mM) for 24 h, the migration area (area% of per field of view) of different groups was evaluated by ImageJ. **D** SK-Hep1 cells with or without stable ZNF638 knockdown, stable USP7 knockdown, P22077 treatment (10 μM), and C75 treatment (2.5 μg/ml) were cultured with or without fructose (8 mM) for 48 h in transwell chambers; the invaded cells were calculated and analyzed by ImageJ. **E** The overall survival difference between high and low expression of USP7 or ZNF638 in HCC patients was analyzed using the transcriptome data from TCGA; the cutoff value was computed by survminer R package. **F** The discrepancy in the ratio of HCC patients with different USP7/ZNF638 expression from different etiologies was analyzed based on the data from TCGA. **G** USP7 and ZNF638 expression were evaluated in four paired HCC paraffin sections (2 of steatosis, 2 of non-steatosis) by immunochemistry. The representative statistical results were performed using one- or two-way ANOVA test and shown as means ± SEM from three independent experiments. *p < 0.05, **p < 0.01, ***p < 0.001, ****p < 0.0001.

It is known that many liver diseases such as hepatic carcinoma attribute to the abnormal lipogenesis. Considering the involvement of USP7 in DNL and oncogenesis, we postulate that USP7/ZNF638 may potentially participate in hepatic carcinogenesis through mediating DNL. As the initiator of hepatic DNL, fructose was found to induce proliferation and colony formation of SK-Hep1 cells (Fig. 7A and Supplementary Fig. S6A). Whereas depletion of ZNF638 or USP7 was efficient in abrogation of the fructose-derived such effects (Fig. 7A and Supplementary Fig. S6A). Using an organoid model containing 70% SK-Hep1 and 30% LXR cells, functional loss of ZNF638 or USP7 has been shown to block fructose-induced organoid growth (Fig. 7B and Supplementary Fig. S6B). In addition, interference of ZNF638 or USP7 was able to inhibit migration and invasion of fructose-induced tumor cells (Fig. 7C, D and Supplementary Fig. S6C). The FASN inhibitor (C75) also efficiently blocked the promoting effects of fructose (Fig. 7A–D and Supplementary Fig. S6A, B, C). Subsequent analysis of the transcriptome data from TCGA revealed that high expression of ZNF638 and USP7 in HCC patients correlated with poor outcomes (Fig. 7E). A comparison between fatty- and virus-related HCC patients showed that higher percentage of USP7 + , ZNF638 + , or double-positive patients occurred in fatty-related groups, implying a key role of USP7 and ZNF638 in fatty-associated HCC (Fig. 7F). To fully elucidate clinical relevance of USP7 and ZNF638, we immunostained the HCC specimens with or without liver steatosis. Although higher expression of both proteins was observed in tumor tissues comparing with para-tumor tissues, the expression of USP7 and ZNF638 was further increased in patients with liver steatosis, and in these patients, USP7 favored nuclear localization (Fig. 7G). To statistically examine the difference, we included additional twelve HCC tissues (six with steatosis and six without steatosis) for immunoblotting with quantification. Similar results were observed, confirming the higher abundance of USP7 and ZNF638 in steatosis-related HCC (Supplementary Fig. S6D). Taken together, USP7/ZNF638 axis plays crucial roles in lipogenesis-associated HCC.

## Discussion

Dysregulated-DNL leading to excessive hepatic fat accumulation is a common feature in many liver diseases. Excessive lipids and FAs resulting from DNL have been proved to cause hepatocellular damages through modification of intracellular organelles or activation of inflammatory pathways^26,27^. Such chronic actions inevitably promote differentiation of satellite cells to myofibroblasts, thereby facilitating the progression of fibrosis^28^, which in turn increases the risk for the development of hepatocellular carcinoma^29^. In addition, aberrant fatty acid synthesis also yields lipid components, which play important roles in membrane structural integrity and tumorigenesis. For example, sphingolipid deriving from fatty acid not only forms the building block of cancer cells, but also suppresses the anti-tumor effect of ceramide^30^. Glycerophospholipid supports mitochondrial membrane synthesis and fulfills the increasing energy requirement of tumor cells^31^. Here, we identified a novel mechanism to induce abnormal DNL via deubiquitinating enzyme USP7, which acted as a key regulator for orchestrating the transcriptional program in promoting hepatic DNL.

Carbohydrates are regarded as the leading risk factors of hepatic DNL among dietary components. Unlike glucose that is metabolized by the cells of the whole body, fructose is predominantly metabolized in liver and shows a more prominent competence in inducing liver steatosis^32^. Intracellular fructose is converted to pyruvate that in turn enters TCA cycle to form citrate (the raw material of DNL), thus initiating DNL. Alternatively, it alters the gene expression related to DNL (e.g., activation of ACACA, FASN, SCD, and SREBP1C), as well as FAs beta oxidation (e.g., suppression of PPARα)^33^. As a result, dietary fructose consumption is closely associated with the development and progression of NAFLD^34,35^, NASH, and spontaneous HCC^36^. Interestingly, autonomous activation of DNL happens in many tumor cells including HCC, owing to the massive needs of energy and membrane formation. As an important pathway involved in hepatic DNL, AKT-mTORC1-S6K axis has been found to be essential for the processing of SREBP1C. SREBP1C predominantly resides in ER binding with SREBP cleavage activating protein (SCAP). Upon stimulation by insulin or nutrients, it transports to Golgi undergoing proteolytic cleavage to form cleaved-SREBP1C, followed by nuclear translocation to initiate DNL. Such process is precisely controlled by AKT-mTORC1 and the downstream effector S6K^37–39^. In the present study, we have identified a novel mechanism of SREBP1C processing under the regulation of USP7 and ZNF638.

**Fig. 8:**
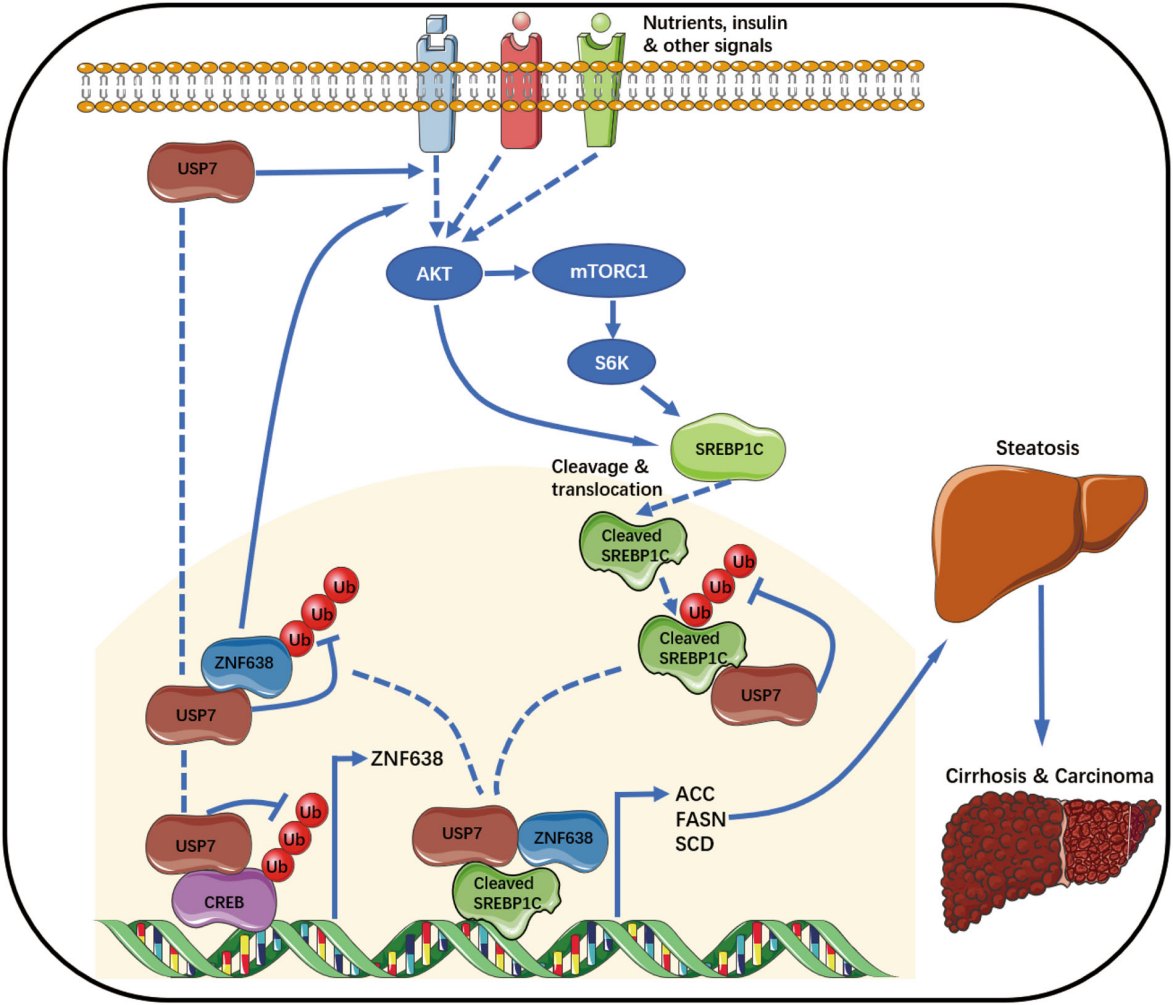
A schematic model illustrating the role of USP7 and ZNF638 in hepatic DNL and diseases. By transcriptional activation of ZNF638 through stabilizing CREB, and deubiquitination of ZNF638, USP7 activates AKT-mTORC1-S6K pathway, thereafter promoting the cleavage and nuclear translocation of SREBP1C; In another hand, USP7 stabilizes cleaved-SREBP1C and forms a USP7-ZNF638-cleaved-SREBP1C complex in cell nucleus. The multi-regulatory mechanisms guarantee the fully activation of SREBP1C as well as its target genes, leading to the enhancement of hepatic DNL and relevant diseases.

In this study, we have unveiled an unusual regulatory role of USP7 in activation of ZNF638. As a DUB enzyme, USP7 not only binds to and stabilizes ZNF638 at protein level, but it simultaneously promotes the transcription of ZNF638 through stabilizing its transcription factor CREB. In fructose-induced lipid accumulation models, USP7/ZNF638 axis has been shown to regulate lipogenesis through facilitating the expression of DNL key enzymes, including ACACA, FASN, and SCD. USP7 or ZNF638 controls the DNL transcription without affecting the abundance of full-length SREBP1C. Instead, they bind to SREBP1C and promote its cleavage through activating AKT-mTORC1-S6K pathway. ZNF638 was also found to be co-localized with SREBP1C on the promoters of target genes (FASN, SCD). Moreover, after cleavage, the nuclear activated isoform of SREBP1C (cleaved-SREBP1C) could be directly stabilized by USP7 independent of AKT-mTORC1-S6K. (Fig. 8). As ZNF638 has been reported to induce the activation of PPARγ, which in turn augments the expression of P85 subunit of phosphatidylinositol-3-kinas (PI3K) in adipocytes^40^, we assume that the promoting effects of USP7/ZNF638 axis on AKT-mTORC1-S6K signaling can be partially mediated via the activation of PPARγ. Thus, USP7 and ZNF638 are robust agonists that participate in multiple steps to control the DNL network. Reasonably, USP7/ZNF638 was also abundantly detected in HCC patients, especially those with liver steatosis, suggesting the central role of USP7 or ZNF638 in DNL-related carcinogenesis. However, as to whether USP7/ZNF638 participates in naturally progressed evolution from lipogenesis to HCC, a long-term mice model with NASH and spontaneous HCC (high-fat diet with glucose and fructose) requires to be further discussed.

In conclusion, our studies uncover an important functional regulatory network among USP7, ZNF638 and cleaved-SREBP1C in hepatic DNL. The novel findings partially explain the molecular mechanisms that lead to abnormal DNL in liver diseases and might provide alternative therapeutical targets for lipogenesis-associated hepatic diseases.

## Materials and methods

### Stable cell lines establishment

Human SK-Hep1, Huh-7, and NH3T3 cells were brought from ATCC. Dulbecco’s modified Eagle’s medium (High glucose, HyClone) added with 10% fetal bovine serum (Gibco), 1% Penicillin–Streptomycin (NCM Biotech) was applied for cell culturing. To stable knockdown the expression of USP7 and ZNF638, SK-Hep1 cells were transfected with USP7Sg-1, USP7Sg-2, ShZNF638 plasmids or control vector plasmids by Lipofectamine 2000 (Invitrogen). Forty-eight hours later, 2 µg/ml of puromycin (Invitrogen) was added to the medium and further maintained for 14 days until the stable cell lines were established. The knockdown efficiency was confirmed by RT-PCR and IB.

### Plasmids, Sh-RNAs, SgRNAs, SiRNAs, GalNAc-conjugated-SiRNAs

Plasmids coding for USP7 (pCDNA3.1-FLAG-USP7), USP7 TRAF domain (P3xFLAG-CMV-10-TRAF), USP7 CD domain (P3xFLAG-CMV-10-CD), USP7 HUBL domain (P3xFLAG-CMV-10-HUBL), wild type ubiquitin (pCDEF-HA-ubiquitin), mutant ubiquitin (pCDEF-HA-K48 ubiquitin, pCDEF-HA-K63 ubiquitin, pCDEF-HA-K48R ubiquitin), Sh-RNA/SiRNA/GalNAc-conjugated-SiRNA targeting ZNF638 and control Sh/SiRNA were designed and purchased from TransheepBio-Tech CO, LTD (Shanghai, China). pGL3-Basic-FASN promoter (−2000~+100), pGL3-Basic-SCD promoter (−2000∼+100), pGL3-Basic-ACACA promoter (−2000∼+100) luciferase plasmids and pRL-TK plasmid were designed and brought from PPL (Public Protein/Plasmid Library, China). Two SgRNAs were designed to knock out the potential sequence of USP7. The authenticity of all the plasmids was verified by DNA sequencing. All transfections were conducted using Lipofectamine 2000 (Invitrogen) following the instructions. The oligos used were listed as follow: Human-ShZNF638-F gatccGCACAGAACAAAGAGGTGAAGTCAAGAGCTTCACCTCTTTGTTCTGTGCtttttg; Human-ShZNF638-R aattcaaaaaGCACAGAACAAAGAGGTGAAGCTCTTGACTTCACCTCTTTGTTCTGTGCg; Mouse-SiZNF638-1-F GCCACAGAACAAAGAAAUGAATT; Mouse-SiZNF638-1-R UUCAUUUCUUUGUUCUGUGGCTT; Mouse-SiZNF638-2-F GAGGGAUAUAAGAAUGCGAAATT; Mouse-SiZNF638-2-R UUUCGCAUUCUUAUAUCCCUCTT; Mouse-SiZNF638-3-F GUACGUAUUUAUGAUCCUGAATT; Mouse-SiZNF638-3-R UUCAGGAUCAUAAAUACGUACTT; Mouse-SiZNF638-4-F CCAGGUACCCUUUACAUUUGATT; Mouse-SiZNF638-4-R UCAAAUGUAAAGGGUACCUGGTT; Human-SgUSP7-1 F-ccgGGGAATGTGGCCCTGAGTGA R-aacTCACTCAGGGCCACATTCCC; Human-SgUSP7-2 F-ccgGGTGTTGTGTCCATCACTCA R-aacTGAGTGATGGACACAACACC.

### RNA extraction, quantitative real-time PCR, luciferase reporter, and ChIP assays

Cells were lysed by Trizol (Beyotime), and the RNA was isolated using Total RNA Isolation Kit (Invitrogen). Reverse transcription was conducted by using BeyoRT^TM^II First Strand complementary DNA Synthesis Kit (Beyotime). Quantitative real-time PCR was performed on LightCycler480II (Roche) system with SYBR green mix kit (Vazyme). The relative Ct values of all RT-PCR results were shown by normalizing the control values to 1 and statically analyzed by GraphPad Prism version 7. The primers used were listed as follow: ZNF638: F-ATGTCGAGACCCAGGTTTAATCC R-TGTGGCCCCATGTTCTGATAA; FASN: F-AAGGACCTGTCTAGGTTTGATGC R-AAGGACCTGTCTAGGTTTGATGC; SCD: F-TCTAGCTCCTATACCACCACCA R-TCGTCTCCAACTTATCTCCTCC; SREBF1: F-GCCCCTGTAACGACCACTG R-GCCCCTGTAACGACCACTG; ACACA F-TATTGCGGCCAATGTCTTTGC R-CACTGGAGTGATAGACTCAACCA. For dual luciferase reporter assay, SK-Hep1 cells with stable USP7 knockdown, ZNF638 knock down, or P22077 treatment (Selleck, 20 μM 24 h) were co-transfected with the plasmids of pGL3-Basic-promoter luciferase and pRL-TK luciferase. Twenty-four hours later, cells were lysed and the luciferase activity was detected by using Dual Luciferase Assay System (Promega). ChIP assays were performed using ChIP assay kit (P2078, Beyotime) according to the manufacturer’s instructions. Briefly, SK-Hep1 cells were incubated with 1% formaldehyde for the DNA-protein crosslinking. After that, cells were collected, lysed, sonicated, and immunoprecipitated with 2 μg of anti-ZNF638, anti-SREBP1C or negative control IgG antibodies. The protein A-agarose beads were used to pull down the DNA-protein-antibody compounds, and the DNA was retrieved by crosslinking reversion and elution with 5 M NaCl. TWo microliters of DNA were used for PCR. The primers used for ChIP were as follows: FASN F-CGCTCCTCAGTCCCAGCCCCA R-GGGCCGCTGCCGTCTCTCTG; SCD F-GGGCCGCTGCCGTCTCTCTG R- CTGGGGAAATGCTAATGAGG.

### Immunoblotting and immunoprecipitation

Liver tissues and hepatoma cells were lysed by RIPA lysis buffer (P0013B, Beyotime) with protease inhibitors (Roche) and phosphatase inhibitors (Roche). All samples were centrifuged at 12,000 rpm, 5 min, 4 °C. Then, the lysates were boiled with SDS Loading Buffer (Beyotime). Protein was isolated by SDS gels and transferred to 0.22 μm PVDF Membranes (Sigma). The membranes were then incubated with primary antibodies, following by washing and probing with secondary antibodies (Southern-Biotech). The blotting was detected using NcmECL Ultra (P10300A/B, NCM Biotech). Primary antibodies used were listed as follow: USP7 (sc-137008, Santa Cruz Biotechnology, 1:500), ZNF638 (A301-548A, Bethyl, 1:1000), Ubiquitin (sc8017, Santa Cruz Biotechnology, 1:500), SCD (sc-515875, Santa Cruz Biotechnology, 1:500), SREBP1C (sc365513, Santa Cruz Biotechnology, 1:500), FASN (C20G5, CST, 1:1000), Phospho-S6k (Thr389) (108D2, CST, 1:1000), S6k (49D7, CST, 1:1000), Phospho-AKT (Ser473) (D9E, CST, 1:1000), AKT (40D4, CST, 1:1000), Acetyl-CoA Carboxylase (ab45174, Abcam, 1:500), phosphor-mTOR (Ser2448) (ab9268, Abcam, 1:1000), mTOR (ab2732, Abcam, 1:1000), Histone3 (ab1791, Abcam, 1:2000), GAPDH (60004-1-Ig, Proteintech, 1:5000), HA (AE008, ABClonal 1:1000). Flag (AE063, ABClonal 1:1000), CREB (A10826, ABClonal 1:1000), MDM2 (SC965, Santa Cruz Biotechnology, 1:500). Each IB assay was repeated at least through three independent experiments, and some of the results were quantified and statistically analyzed via ImageJ and GraphPad Prism software. For immunoprecipitation, cells were lysed by RIPA lysis buffer (P0013C, Beyotime) containing protease inhibitors. For the nuclear immunoprecipitation, cell nuclear extracts were firstly harvested using Nuclear and Cytoplasmic Protein Extraction Kit (Beyotime). Isolated nuclear lysates were firstly incubated with protein A/G agarose (Bioepitope) for preclearing, then immunoprecipitated with the primary antibodies overnight following by the incubation with protein A/G agarose for 2.5 h. The precipitates were eluted and subjected to SDS gels for IB assays.

### Ubiquitination and protein stability assay

For the ubiquitination assays, stable or P22077 treated (Selleck, 20 μM 24 h) cells were transiently transfected with or without the wild type or mutant Ubiquitin plasmids. Eight hours before collecting, 100 nM of Bortezomib (Sigma) was added for the suppression of proteasome-dependent protein degradation. Then, cells were collected and lysed for IP and IB assays. For the protein rescue assay, Bortezomib (Sigma, 100 nM) was added in USP7 knockdown or P22077 treated (Selleck, 20 μM 24 h) cells 8 h before harvesting. For the protein half-life assay, cells were administrated with CHX (100 μg/ml, C7698, Sigma) and collected at the indicated intervals. IB was used to determine the protein attenuation; quantitative analysis was performed by ImageJ based on the relative protein expression normalized to GAPDH.

### Immunochemistry, immunofluorescence, and HE staining

For the immunochemistry assay, paraffin sections of paired human HCC or mouse liver were deparaffinized, rehydrated, following by antigen retrieval in sodium citrate buffer. After 1 h of blocking by 1% BSA at room temperature, the sections were then sequentially incubated with the indicated primary and secondary antibodies. DAB staining was performed for the final detection. For the cell immunofluorescence assay, cells were fixed and permeabilized by 4% formaldehyde and 0.25% Triton X-100, then blocked with 1% BSA for 1 h at room temperature. After 12 h of incubation with the primary antibodies diluted with 1% BSA, cells were washed and probed with Fluor–labeled secondary antibodies (ABclonal Technology) and DAPI (CST), following by visualization via confocal laser-scanning microscope or fluorescence microscope. Hematein and eosin staining were used to determine the pathological changes in the mice sections.

### Oil-Red-O assay and triglyceride detection

For assessing the lipids accumulation, Oil-Red O Kit (Meilun Biotechnology) was used for staining in cells and frozen sections of mouse liver tissues. For TG content detection, cells or liver tissues were collected and sonicated (300 W, 3–5 s a cycle, 30 s interval). The supernatant TG content (mmol/gprot) was then measured by TG kit (Nanjing Jiancheng Biology Engineering Institute) at the absorbance of 450 nm according to manufacturer’s instructions.

### CCK-8, clone formation, and organoid growth assay

For detecting the growth effect of fructose, SK-Hep1 cells were treated with different concentrations of fructose (2, 4, 8 mM) for different time points (24, 48, 72 h). For evaluating the role of ZNF638 and USP7 on lipogenesis-related cell growth, cells with ZNF638 or USP7 knockdown, P22077(Selleck, 10 μM) and C75(C5490, Sigma, 2.5 mg/ml) treatment were planted in 96-well plate (5000 cells per well) and cultured with or without fructose (8 mM) for 24 or 48 h. Cell Counting Kit-8(E606335, Sangon Biotech) was used to analyze cell viability under the absorbance of 450 nm. For the clone formation assay, SK-Hep1 cells with or without stable ZNF638 or USP7 knockdown were plated at a density of 1000 cells/well in 6-well plates and treated with or without fructose (8 mM) for 2 weeks. Some groups of cells were treated with P22077(Selleck, 5 μM) and C75 (C5490, Sigma, 1 mg/ml) three days after the starting date. Cell clones were collected and fixed with 4% paraformaldehyde for1h at room temperature, stained with crystal violet (C0121, Beyotime) and calculated by ImageJ. For the organoid growth assay, 70% of different SK-Hep1 cells (ZNF638 knockdown, USP7 knockdown, treatment of 5 μM P22077, treatment of 1 μg/ml C75) and 30% of LXR cells were mixed and glued with Collagen I (MB5680, Meilun Biotechnology, 5 μg/ml); the culture medium with or without fructose (8 mM) was added with 20 ng/ml b-FGF (MB2458, Meilun Biotechnology), 20 ng/ml HGF (294-HG-005, R&D) to promote the growth of both cell lines. At the time points of 6 h, 3 days and 5 days, the formed organoids were photographed and the volumes were calculated as 1/2 long axis*minor axis^2^.

### Wound healing and transwell assay

For the wound healing test, cells with stable ZNF638/USP7 knockdown, P22077 (Selleck, 10 μM) or C75 (C5490, Sigma, 2.5 mg/ml) treatment were pre-scratched with sterilized 10 μl pipette tips and cultured with or without fructose (8 mM) for 24 h in 6-well plates. Wound healing was observed under the microscope at different time points. For the transwell assay, chambers installed with 8 μm pore size, 6.5 mm diameter polycarbonate membrane (3422, Corning costar) were used to isolate invasive cells. Grouped cells as the wound healing assay were cultured in the upper wells (1 × 105 cells/well), while the bottom wells were filled with condition medium. After incubating for 48 h, the chambers were fixed with 4% paraformaldehyde for 40 min at room temperature, stained with crystal violet (C0121, Beyotime) and counted by ImageJ.

### Bioinformatic analysis

The RNA-seq data (FPKM) and the matched phenotype (overall survival, virus status, and BMI) were fetched from The Cancer Genome Atlas (https://www.cancer.gov/about-nci/organization/ccg/research/structural-genomics/tcga). Only primary tumor samples were included for analysis. The correlation analysis was performed by using cor.test, and the RNA expression was visualized by using ggplot2 in R. We stratify patients into groups according to the median expression of a given gene. The survival package (https://cran.r-project.org/web/packages/survival/index.html) and survminer package (https://cran.r-project.org/web/packages/survminer/index.html) were used to visualize the survival curve in R. The *p*-value was computed via log-rank test.

### Animal model

Male C57BL/6 mice were purchased from Laboratory Animal Center of Nantong University. All mice were maitained at 20 ± 2 °C with humidity of 50 ± 10% and a 12 h light/12 h dark cycle. For the fructose-induced mouse hepatic steatosis model, mice were fed with standard chow diet or diet with 30% fructose (F875004, Macklin) in drinking water. The mice fed with fructose were subclassified and disposed to intraperitoneal injection of P22077(Selleck, 10 mg/kg) and GalNAc-SiZNF638 (2 μg/g BW) every week, each group contained five mice. The body weight was measured per week. After 4 weeks of maintaining, mice were sacrificed and the livers were obtained for subsequent assays.

### Patient samples

The paired HCC paraffin and fresh samples were obtained from pathology department of Affiliated Hospital of Nantong University. The human study was approved by the Human Research Ethics Committee of Affiliated Hospital of Nantong University and conducted in accordance with all relevant ethical regulations. Informed consent was obtained from each participant.

### Statistics

Statistical analysis was performed with GraphPad Prism software. The data shown in the figures were representative of three independent experiments and were analyzed by one- or two-way analysis of variance (ANOVA) with Dunnett’s post test or unpaired *t*-test, and *p* < 0.05 was considered statistically significant.

## Supplementary information

Figure S1

Figure S2

Figure S3

Figure S4

Figure S5

Figure S6

Supplemental figure legends
