# Supplementary figures and images for: USP7 mediates pathological hepatic de novo lipogenesis through promoting stabilization and transcription of ZNF638
Source: Cell Death Dis. 2020 Oct 10;11(10):843. doi: 10.1038/s41419-020-03075-8 (PMC7548010; doi:10.1038/s41419-020-03075-8)

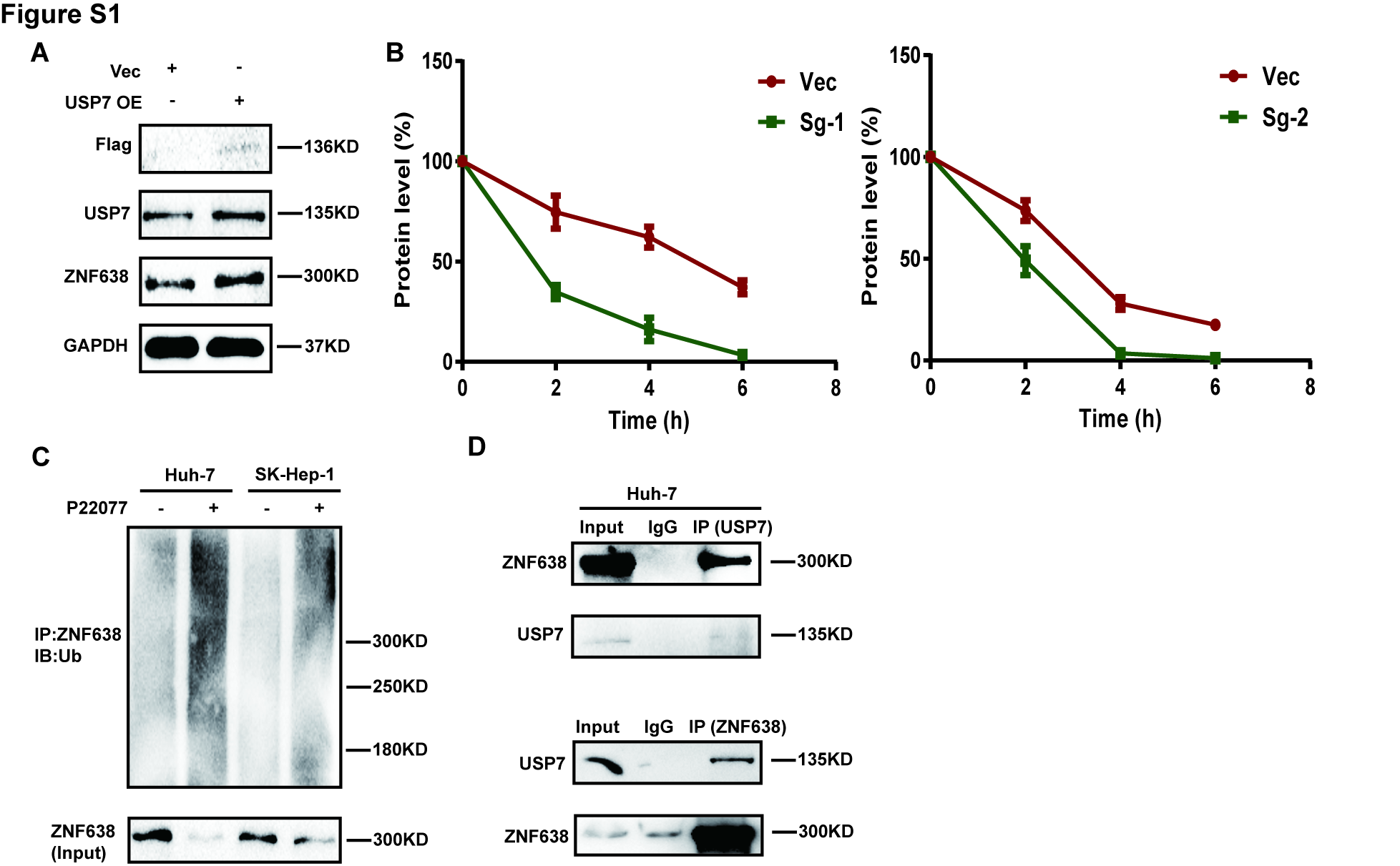

Supplement: Supplementary file 1 — Figure S1 [file 41419_2020_3075_MOESM1_ESM.tif]

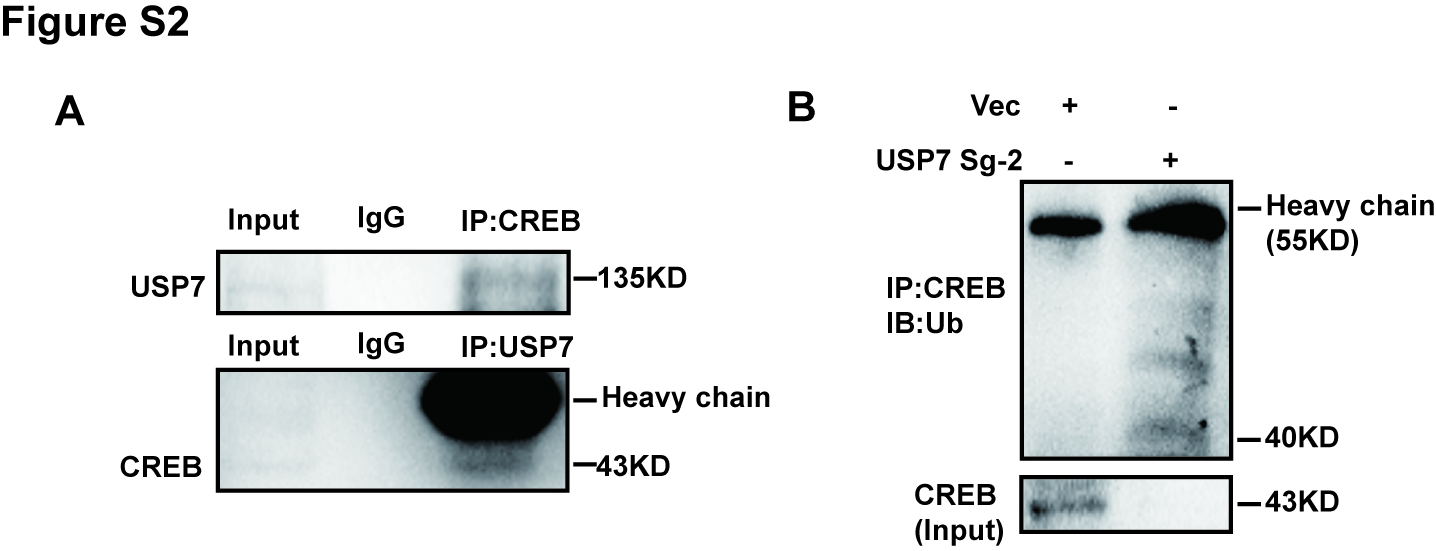

Supplement: Supplementary file 2 — Figure S2 [file 41419_2020_3075_MOESM2_ESM.tif]

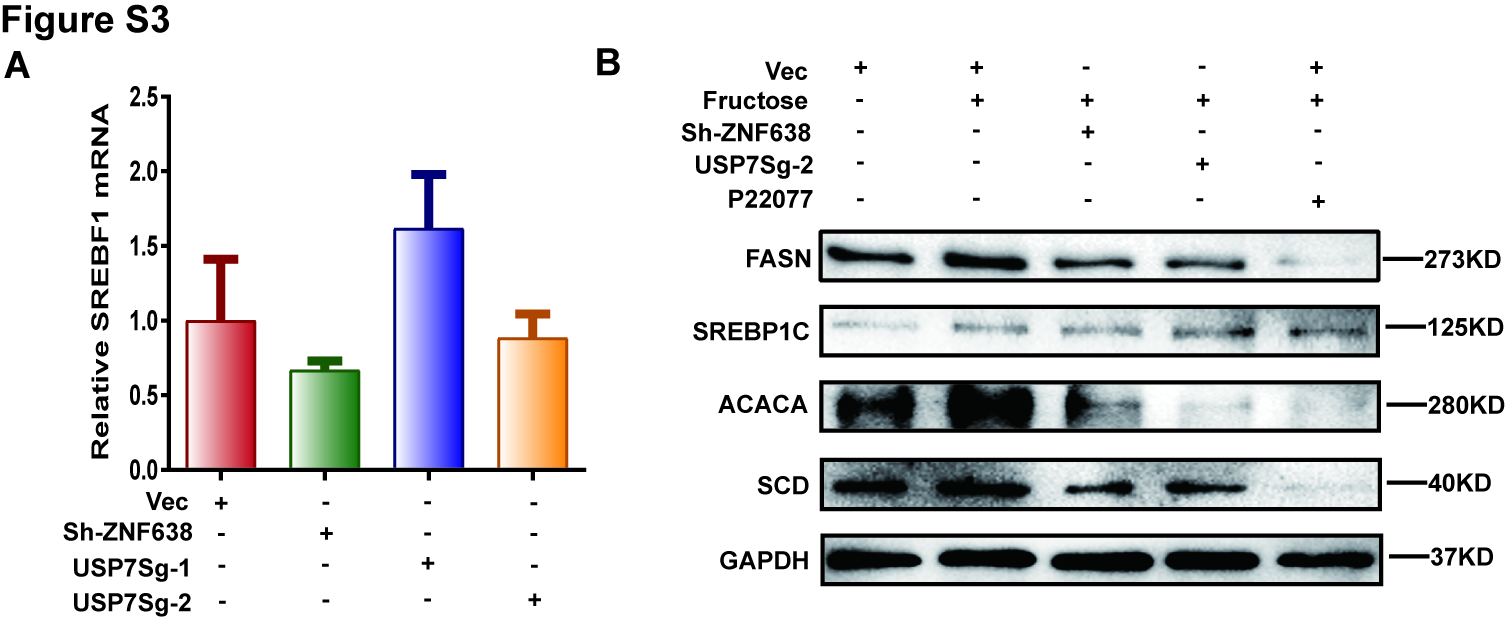

Supplement: Supplementary file 3 — Figure S3 [file 41419_2020_3075_MOESM3_ESM.tif]

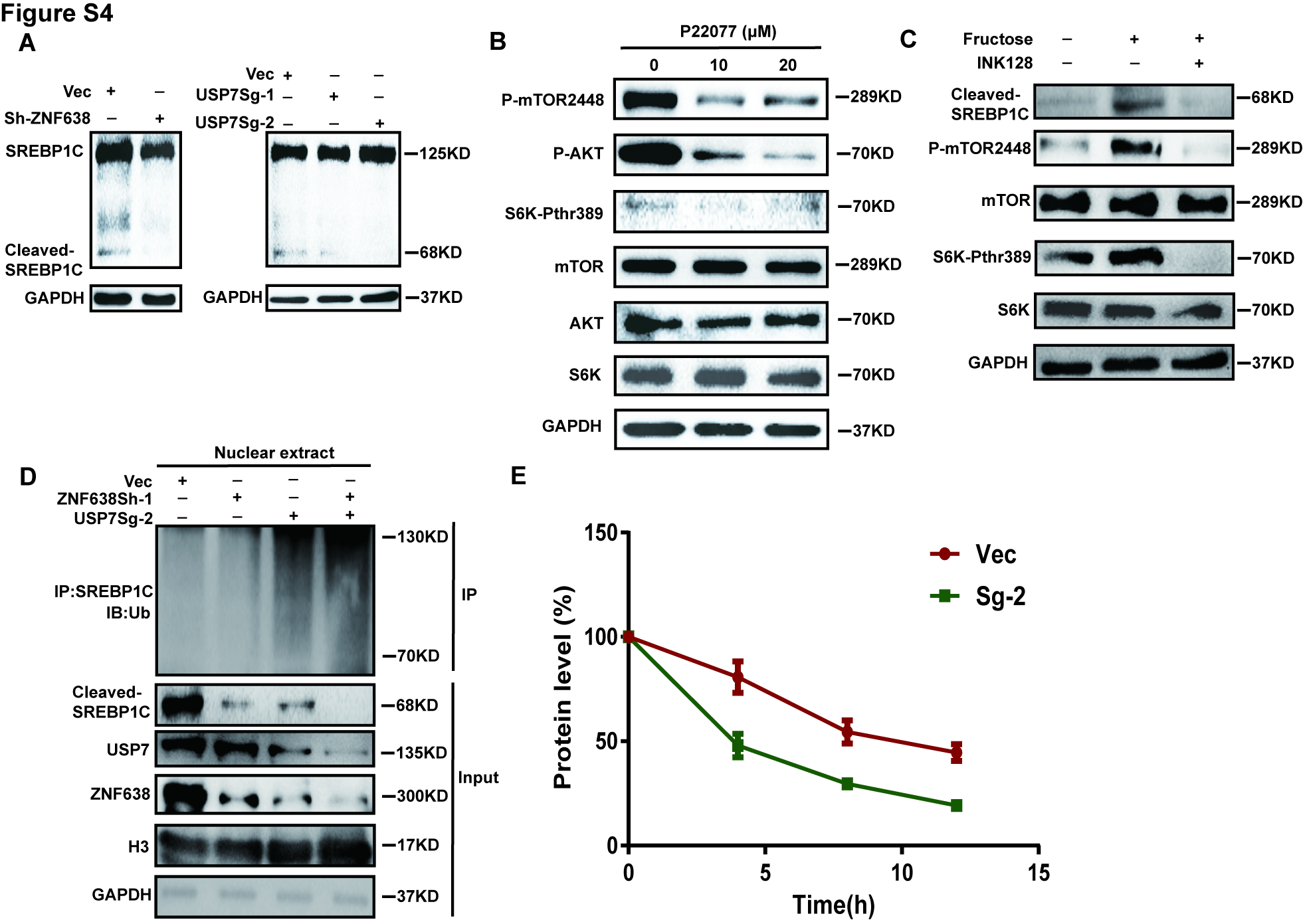

Supplement: Supplementary file 4 — Figure S4 [file 41419_2020_3075_MOESM4_ESM.tif]

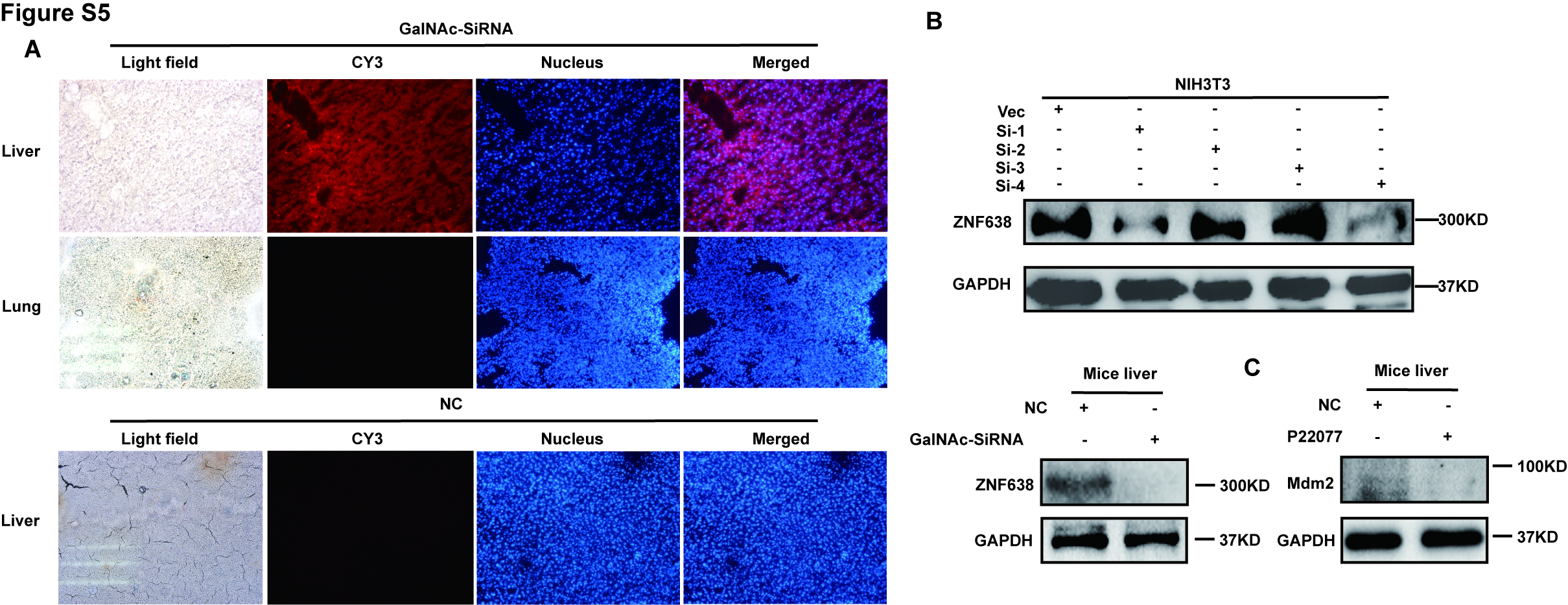

Supplement: Supplementary file 5 — Figure S5 [file 41419_2020_3075_MOESM5_ESM.tif]

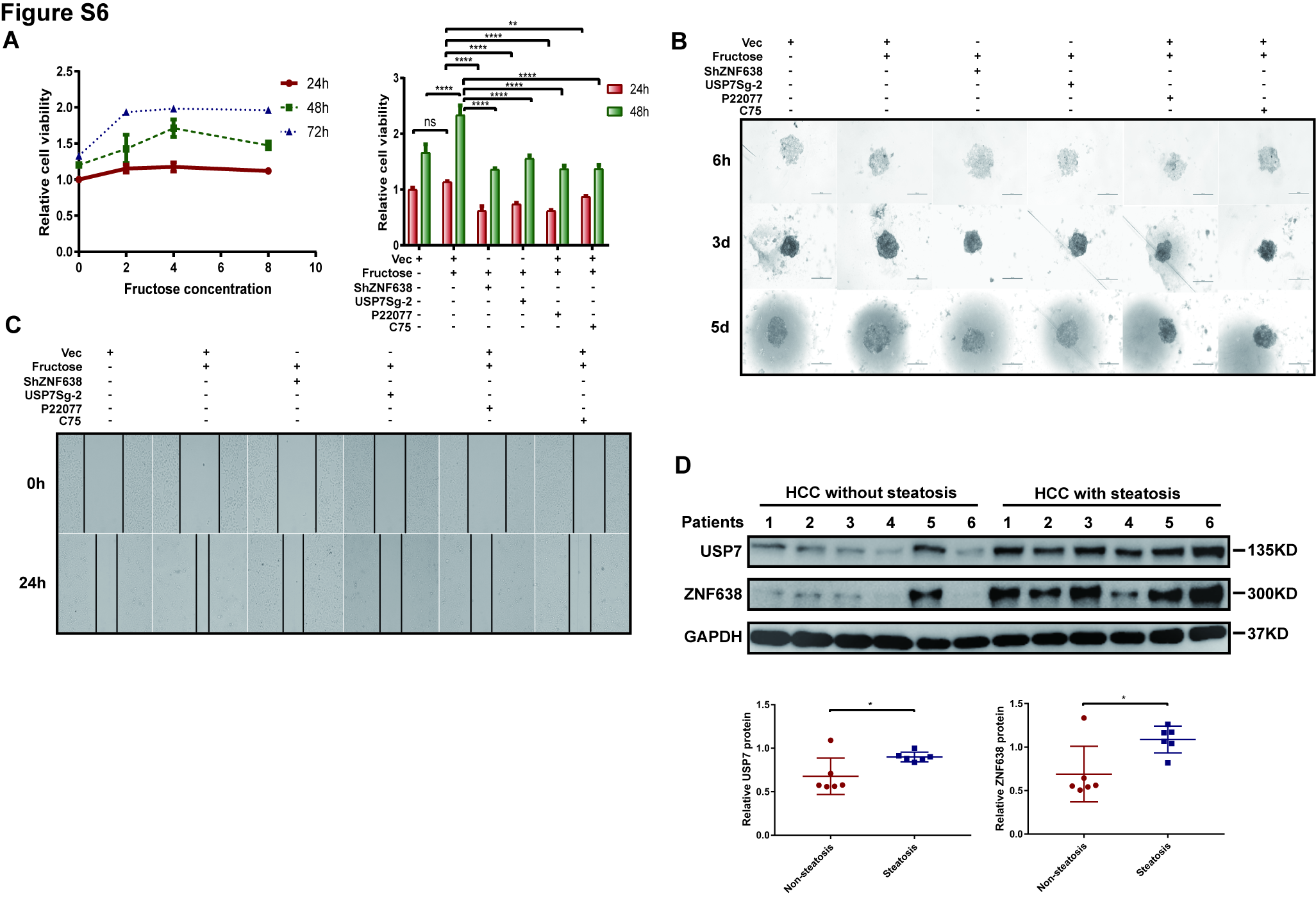

Supplement: Supplementary file 6 — Figure S6 [file 41419_2020_3075_MOESM6_ESM.tif]
