# Supplementary material for: USP7 mediates pathological hepatic de novo lipogenesis through promoting stabilization and transcription of ZNF638
Source: Cell Death Dis. 2020 Oct 10;11(10):843. doi: 10.1038/s41419-020-03075-8 (PMC7548010; doi:10.1038/s41419-020-03075-8)
Supplement: Supplementary file 7 — Supplemental figure legends [file 41419_2020_3075_MOESM7_ESM.docx]

**Figure S1.** (A) Enforced expression of USP7 led to the up-regulation of ZNF638 protein. (B) Quantitative analysis of the ZNF638 protein half-life relative to GAPDH in normal and USP7 deficient (Sg-1, Sg-2) SK-Hep1 cells. (C) P22077 (20μM 24h) treatment significantly increased ubiquitination level of ZNF638 in Huh-7 and SK-Hep1 cells. (D) ZNF638 and USP7 were endogenously interacted with each other in Huh-7 cells as determined by Co-IP assay.

**Figure S2.** (A) The endogenous interaction between USP7 and CREB was detected in SK-Hep1 cells. (B) The ubiquitination levels of CREB in SK-Hep1 cells with or without USP7 knockdown were examined by immunoprecipitation.

**Figure S3.** (A) The relative mRNA of SREBF1 was unaltered in USP7-deficient, ZNF638-deficient SK-Hep1 cells as determined by real-time PCR. (B) Fructose (8mM 48h) induced up-regulation of ACACA, FASN, SCD but not full-length of SREBP1C was attenuated by inhibition of USP7 and ZNF638.

**Figure S4** (A) USP7 or ZNF638 knockdown reduced cleaved-SREBP1C in SK-Hep1 cells. (B) Pharmacological inhibition of USP7 in SK-Hep1 cells reduced phosphorylation levels of mTOR, AKT, and S6K. (C) Fructose (8mM 48h) mediated mTOR activation and Cleaved-SREBP1C induction could be eliminated by mTOR inhibitor INK128 (0.1μM, 24h) in SK-Hep1 cells. (D) Genetic deletion of ZNF638 in USP7 deficient SK-Hep1 cells further inhibited cleaved-SREBP1C but not its ubiquitination. (E) Quantification of the half-life of cleaved-SREBP1C relative to GAPDH in normal and USP7 deficient SK-Hep1 cells was revealed using ImageJ.

**Figure S5** (A) The hepatic delivery efficiency of GalNAc conjugated ZNF638 SiRNA labeled by CY3 was identified by red fluorescence in mice liver. (B) The inhibitory efficiency among four ZNF638 Si-RNAs was determined by immunoblotting in NIH3T3 cells. Subsequently, Si-1 with most favorable suppressive effect in vitro was chosen for in vivo test, and declined protein of ZNF638 was detected in liver with the administration of GalNAc-ZNF638 SiRNA-1. (C) The efficiency of intraperitoneal injection of P22077 was verified by evaluating the protein level of MDM2 in liver.

**Figure S6** (A) SK-Hep1 cells were treated with fructose at different concentration and time points; SK-Hep1 cells with stable ZNF638 knockdown, stable USP7 knockdown, P22077 treatment (10μM), and C75 treatment (2.5μg/ml) were cultured with or without fructose (8mM) for one or two days (right). CCK-8 kit was used to check the cell viability. (B) The image of organoids assay relating to Figure 7B. (C) The image of wound healing assay relating to Figure 7C. (D) Immunoblotting with quantitative analyzing were performed by applying 12 fresh HCC tissues (6 with hepatic steatosis, 6 without steatosis). The representative statistical results were performed using 1- or 2-way ANOVA test, unpaired t test, and shown as means ± SEM from 3 independent experiments. * p<0.05, ** p<0.01, *** p<0.001, **** p<0.0001.
